# Focal adhesion kinase (FAK): emerging target for drug-resistant malignant tumors

**DOI:** 10.1007/s11033-025-10296-7

**Published:** 2025-02-20

**Authors:** Jaya Aakriti, Megh Pravin Vithalkar, Swastika Maity, Krishnaprasad Baby, Prabhakara R. Nagareddy, Yogendra Nayak

**Affiliations:** 1 Department of Pharmacology, Manipal College of Pharmaceutical Sciences, Manipal Academy of Higher Education, Madhava Nagar, Manipal, 576104, India; 2 Department of Internal Medicine, Cardiovascular Section, University of Oklahoma Health Sciences Center (OUHSC), Oklahoma City, OK USA

**Keywords:** Cancer, Focal adhesion kinase, FAK-structure, FAK-inhibitors, Defactinib, Clinical trials, Combination therapy

## Abstract

Malignant tumors associated with drug resistance present a significant challenge for clinicians and drug developers. Mutations and alterations within the tumor microenvironment frequently drive cancer cell invasion and metastasis. Despite identifying numerous molecular targets and lead compounds, achieving sustained efficacy remains challenging due to the rapid mutation rates and the emergence of resistance. Recently, Focal Adhesion Kinase (FAK), a non-receptor tyrosine kinase, has emerged as a promising target for inhibiting cancer progression, with several lead molecules advancing through clinical trials. FAK plays a critical role in cancer pathology by regulating cell adhesion, migration, proliferation, and survival. Its structure comprises three domains- the N-terminal FERM domain, kinase domain, and C-terminal focal adhesion targeting domain- contributing to its functional versatility. Ligands targeting the FERM and kinase domains can suppress cancer cell proliferation, invasion, and migration. The FERM domain, a member of the ezrin, radixin, and moesin family, is particularly noteworthy for its ligand-binding capabilities and potential to inhibit tumor growth. While FAK is a compelling anticancer target, challenges such as tissue-specific physiological variability and broad ligand specificity remain. This review provides a detailed analysis of FAK’s role in cancer progression and explores emerging molecules targeting FAK as potential treatments for drug-resistant malignant tumors.

**Graphical Abstract:**

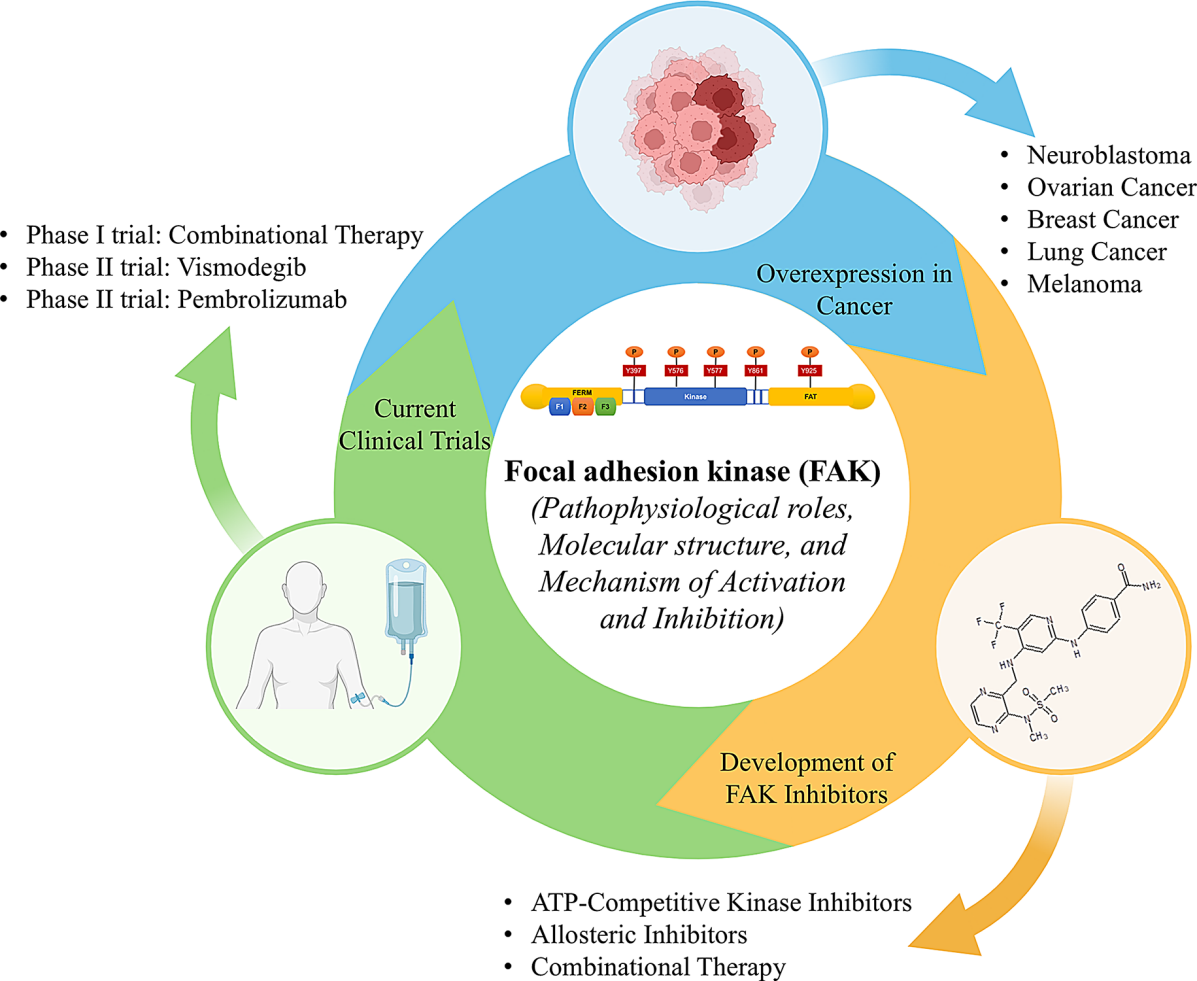

## Introduction

Malignant tumors are challenging to treat due to their high mutation rates and resistance to therapy [1]. This has led to the rise of molecular-targeted therapy, which aims to overcome resistance mechanisms. These therapies primarily focus on disrupting cancer cell signaling pathways or targeting components within the tumor microenvironment [2]. Among these, targeting signaling pathways has emerged as a prominent approach. Established drug targets include Bcl-2, Bax, CDK, EGFR/ErbB1, ERK, FGFR, HER2/ErbB2, HGF, mTOR, MAPK, MAPK/ERK kinase, PARP, PI3K, RAF, and BRAF [3]. Similarly, drug targets within the tumor microenvironment include CTLA-4, FGFR, MHC, PD-1, PD-L1, PDGFR, TCR, and VEGFR [4].

Focal Adhesion Kinase (FAK) has recently gained prominence as a crucial therapeutic target due to its potential to impede cancer progression. FAK, a 125-kDa signaling protein first identified in 1992 from chicken embryos, was initially detected using monoclonal antibodies like mAb,2a7 [5]. Known as pp125, this protein demonstrated increased phosphorylation in the presence of pp60^v-src^ [6], and its amino acid sequence confirmed it as a cytoplasmic protein tyrosine kinase [7].

The discovery of human FAK in 1996 advanced our understanding of its roles in normal and cancer cells, including its various phosphorylation sites involved in signal transduction [8]. It was observed that FAK is critical in regulating cell adhesion, proliferation, migration, and survival [9]. Its primary phosphorylation site, tyrosine-397 (Y397), provides a binding site for the SH2 domain of Src, leading to the activation and overexpression of FAK, which drives tumorigenesis [10]. Cancer-associated proteins such as Ephrin receptor A2, Aurora-A, and FAK have been identified as critical targets for cancer therapy, with FAK being implicated in epigenetic modifications, genomic instability, and epithelial-mesenchymal transition, all of which contribute to metastasis [11].

The crystal structure of FAK, resolved in 2002 using nano-volume droplet techniques, provided insights into its structural basis for drug design [12]. In 2008, Pfizer developed PF-00562271, a FAK inhibitor targeting the ATP binding site of its kinase domain, demonstrating an IC_50_ of 1.5 nmol/L in tumors expressing FAK [13]. Major pharmaceutical companies, including Pfizer, Novartis, GlaxoSmithKline, and others, have invested in developing FAK inhibitors and hold patents for related molecules [14]. Thus, targeting FAK holds substantial promise for developing improved anticancer therapies. This review highlights the current trends in FAK research, focusing on its implications in cancer progression and the latest developments in several FAK-targeting molecules, including defactinib, VS-4718, BI-853520, and GSK2256098, that are progressing through various clinical and preclinical stages, for therapeutic applications.

## Methodology

### Database selection and search strategy

A comprehensive literature review was conducted using key scientific databases, including Scopus, PubMed, Web of Science, and Google Scholar, to identify relevant studies on FAK as a therapeutic target in cancer. Search terms such as “Focal Adhesion Kinase,” “FAK inhibitors,” “cancer metastasis,” “drug resistance,” “FAK domains,” and “FERM domain inhibitors” were employed, utilizing Boolean operators (AND, OR) to refine and optimize search results.

### Criteria for inclusion and exclusion

Studies were included if they focused on FAK’s role in cancer progression, its involvement in molecular pathways regulating cell adhesion, migration, proliferation, and survival, or novel FAK-targeting molecules in clinical or preclinical development. Preference was given to pioneering research and studies published in peer-reviewed journals within the last ten years, particularly those investigating FAK inhibition in drug-resistant cancer models. Excluded studies were those without experimental data on FAK, those centered on non-cancer-related pathways of FAK, or publications in languages other than English. Review articles without primary data were also omitted.

### Screening and data extraction

The review process involved an initial screening of titles and abstracts for relevance, followed by a detailed assessment of full-text articles that met the inclusion criteria. Critical data were extracted from each selected study, including information on examined FAK inhibitors, specific domains targeted (FERM or kinase domains), cellular and animal models used, and findings on their efficacy in inhibiting cancer cell proliferation, migration, and invasion. Particular attention was given to studies highlighting FAK inhibition as a strategy to counteract drug resistance in cancer cells.

### Data synthesis and analysis

The extracted data were synthesized to provide a comprehensive overview of the therapeutic potential of FAK inhibitors in addressing drug-resistant cancers. The review underscores FAK’s multifaceted role in cancer progression and highlights emerging molecular candidates targeting FAK as innovative approaches to mitigate drug resistance and improve therapeutic outcomes in malignant tumors.

## Physiological and pathological roles of FAK

### Cell adhesion

Adherence is essential for tissue development and maintenance, facilitating cell communication and regulatory functions. Cell adhesion refers to the ability of a single cell to bind to another cell or the extracellular matrix (ECM). This process is critical for regulating cell division, the cell cycle, migration, survival, and adhesion. For cell-matrix adhesion, catalytic complexes between Protein Tyrosine Phosphatase 1B (PTP1B) and FAK require intact tyrosine residues Y31 and Y118 on paxillin [15]. Impaired cell adhesion can lead to various diseases, including atherosclerosis and cancer. In malignancies, diminished cell adhesiveness disrupts structural integrity, promoting tumor invasion and metastasis [16].

### Cell migration

FAK plays a pivotal role in cell migration. While the exact mechanisms of FAK-regulated migration remain unclear, it is evident that FAK synchronizes with integrin signaling to ensure proper activation. PTEN, a tumor suppressor protein encoded by the PTEN gene, interacts with FAK to inhibit migration through dephosphorylation [17].

### Eliminating pathogens

Neutrophils serve as the first line of defense against pathogens, employing enzymatic degradation and phagocytosis to protect the host. Neutrophils rely on integrin receptors to facilitate proper contact with the ECM for effective migration. A deficiency in the integrin beta chain-2 results in leukocyte adhesion deficiency type-1. Integrin-mediated FAK activation is a critical step for neutrophil migration [18].

### Regulation of beta-cells

FAK is a connector between the ECM and the cytoplasmic actin network. The actin cytoskeleton plays a key role in insulin secretion. Preclinical studies reveal that FAK-deficient β-cells exhibit impaired insulin secretion, highlighting FAK’s critical role in insulin signaling, pancreatic β-cell viability, and function by regulating actin dynamics and granule trafficking [19].

### Autophagy

The interaction between FAK and the ARF tumor suppressor protein negatively regulates cancer cells via p53-dependent and p53-independent pathways. ARF, localized in the nucleolus, is responsible for arresting the cell cycle at the G1 and G2 phases. FAK and ARF contribute to the autophagy of cancer cells, demonstrating their combined role in cellular regulation [20].

## FAK overexpression in cancers

### Neuroblastoma

The phosphorylation of Y397 in FAK is significantly overexpressed in neuroblastoma. Okadaic acid, a serine phosphatase inhibitor, induces focal adhesion loss by inhibiting phosphorylation at Y397, thereby preventing FAK activation. Studies indicate that FAK is overexpressed in 73% of neuroblastoma tumor samples [21]. In murine models, FAK has been identified as a key regulator of neuroblastoma cell migration and metastasis through its interaction with Neogenin-1, which promotes FAK autophosphorylation and activates integrin β1 [22]. Furthermore, the FAK-Src-Paxillin signaling axis has been established as a prognostic marker for poor outcomes in human neuroblastoma patients, highlighting its potential as a therapeutic target. This signaling pathway underscores FAK’s pivotal role in driving neuroblastoma metastasis [23]. Therefore, FAK represents a promising druggable target for future neuroblastoma therapies.

### Breast cancer

FAK has emerged as a promising therapeutic target in breast cancer, with clinical trials of FAK inhibitors yielding encouraging results, particularly in combination therapies aimed at enhancing the efficacy of other anticancer agents [24]. Additionally, FAK has been implicated in promoting vasculogenic mimicry in metastatic breast cancer cells. Elevated integrin-linked kinase and FAK expression have been linked to an enhanced capacity for these cells to form vascular-like structures, further supporting its role in cancer [25].

FAK is also involved in estrogenic signaling pathways in breast cancer. The G-protein coupled estrogen receptor (GPER) has been shown to stimulate breast cancer metastasis. Studies in triple-negative breast cancer (TNBC) cell lines, such as MDA-MB-231 and SUM159, have demonstrated that FAK is a critical mediator of GPER signaling. Inhibition of FAK successfully prevented GPER-induced TNBC cell migration, underscoring the role of FAK in GPER-driven metastasis [26]. These findings collectively highlight FAK as a multifaceted therapeutic target in breast cancer, with potential applications across diverse molecular and cellular mechanisms driving tumor progression and metastasis.

### Ovarian cancer

FAK inhibitors have emerged as promising therapeutic agents for ovarian cancer by targeting critical signaling pathways involved in tumor progression. Compounds such as compound 36, E2, BI-853520, and PF-562271 have demonstrated significant efficacy in preclinical models, effectively reducing tumor growth, migration, and invasion [27]. These inhibitors achieve their therapeutic effects by disrupting FAK-associated signaling pathways, including Src, AKT, and PI3K/AKT/mTOR, reducing cancer cell proliferation and increasing apoptosis. This positions FAK inhibition as a vital strategy in ovarian cancer treatment [27, 28].

The efficacy of FAK inhibitors is further enhanced when combined with conventional chemotherapy agents. For example, the combination of TAE226 with docetaxel significantly reduced tumor burden and prolonged survival in ovarian cancer models [29]. Y15 and vitamin E have also been shown to amplify the cytotoxic effects of cisplatin and paclitaxel, even in chemo-resistant ovarian cancer cell lines [30]. Defactinib (VS-6063), a second-generation FAK inhibitor, has shown considerable promise in clinical trials. It is currently being evaluated in combination with RAF/MEK inhibitors for patients with low-grade serous ovarian cancer. Preliminary results indicate that this combination leads to significant tumor shrinkage and improved overall response rates compared to monotherapy [31].

FAK overexpression in ovarian cancer has also been linked to the activation of the PI3K-AKT pathway and the transcription factor Krüppel-like factor 8 (KLF8), which play roles in regulating the cell cycle and promoting epithelial-to-mesenchymal transition. This highlights the therapeutic potential of drugs that target FAK phosphorylation sites and disrupt focal adhesion assembly, offering a compelling strategy for ovarian cancer treatment [32].

### Renal cancer

Studies have shown that inhibiting FAK, through either genetic knockdown or pharmacological inhibitors, significantly reduces tumor growth and induces apoptosis in clear cell renal cell carcinoma (ccRCC) [33]. Recent developments have led to the creation of novel dual inhibitors that target both FAK and histone deacetylase 2 (HDAC2). These compounds have shown superior efficacy in preclinical models by inhibiting ccRCC growth and proliferation more effectively than standard treatments. They also induce cell cycle arrest and promote apoptosis, highlighting their potential as therapeutic agents [34].

Combining FAK inhibitors with other treatments has demonstrated synergistic effects, enhancing their antitumor efficacy. For example, the combination of saracatinib, a Src kinase inhibitor, with sunitinib, a receptor tyrosine kinase (RTK) inhibitor, significantly inhibited cell migration and growth in renal cancer models [35]. Moreover, research has indicated that dual inhibition of FAK and platelet-derived growth factor receptor-beta (PDGFR-β) synergistically decreases the survival of primary Wilms tumor cells, suggesting potential applications in pediatric renal tumors [36].

In ccRCC, lysyl oxidase-like-2 (LOXL2) induces metastatic changes, such as epithelial-to-mesenchymal transition (EMT), through the steroid receptor coactivator (Src)/FAK signaling pathway. Studies comparing metastatic and non-metastatic kidney cancers have reported a 2 to 2.5-fold overexpression of FAK and paxillin mRNA in metastatic cases, further reinforcing the critical role of FAK in promoting metastasis in ccRCC [37].

### Melanoma

The Y925F mutation in FAK has been shown to suppress metastasis in melanoma models by downregulating key pathways, such as Erk phosphorylation and vascular endothelial growth factor (VEGF) expression [38]. FAK inhibitors are also being investigated with other anticancer drugs to enhance therapeutic efficacy and overcome chemotherapy resistance. For instance, inhibiting the FAK-IGF-1R protein interaction with novel small molecules, such as INT2-31, has demonstrated therapeutic potential by disrupting downstream signaling, decreasing cell proliferation, and inducing apoptosis [39]. FAK inhibitor-based combinations with MEK or PKC inhibitors also initiate synergistic antitumor effects in uveal melanoma [40]. Melanoma typically invades nearby lymph nodes, where FAK signaling stimulates the expression of vascular cell adhesion molecule-1 (VCAM-1). The FAK inhibitor PF-271 has been shown to significantly decrease VCAM-1 level, thereby reducing B16F10 melanoma cell adhesion and transmigration. Furthermore, oral administration of PF-271 has reduced cervical, inguinal, and popliteal VCAM-1 expression in C57BL/6 mice, supporting the potential use of FAK inhibitors in melanoma treatment [41].

### Lung cancer

Studies have shown that in lung adenocarcinoma, FAK inhibitors, demonstrate limited efficacy as monotherapies, but show significantly enhanced effectiveness when combined with other treatments. For instance, combining FAK and ERK5 inhibitors has led to improved antitumor responses and reduced resistance in KRAS mutant non-small cell lung cancer (NSCLC) [42]. Such combination strategies are crucial for overcoming drug resistance and achieving better therapeutic outcomes. Several FAK inhibitors are currently undergoing clinical trials, with defactinib (VS-6063) emerging as a promising candidate [43]. Beyond directly targeting FAK, these inhibitors also affect other critical signaling pathways involved in cancer progression, such as the PI3K/AKT and MAPK-ERK pathways. This multi-pathway targeting amplifies their therapeutic impact, making FAK inhibitors valuable components of comprehensive cancer treatment strategies [44].

Additionally, FAK inhibitors have been reported to induce cellular senescence in lung cancer cells, potentially by downregulating the expression of enhancer of zeste homolog 2 (EZH2), a protein associated with cancer cell proliferation and survival [45]. One notable FAK inhibitor, Y15, targets the autophosphorylation of FAK at tyrosine 397 (Y397). Preclinical studies have demonstrated its significant antitumor effects both in vitro and in vivo. In lung cancer xenograft models with RAS or EGFR mutations, Y15 effectively inhibited tumor growth, highlighting its potential as a therapeutic agent for NSCLC [46]. Moreover, FAK-mediated laminin-5 overexpression in lung cancer plays a critical role in cell growth, adhesion, and invasion. Thus, targeting both laminins and integrin-FAK signaling could present a potential therapeutic approach for lung cancer [47].

### Pancreatic Cancer

FAK inhibitors have shown considerable promise in preclinical pancreatic ductal adenocarcinoma (PDAC) models, targeting both tumor cells and their microenvironment. For example, Y15 targets the Y397 autophosphorylation site of FAK, reducing tumor growth and enhancing sensitivity to gemcitabine in vivo [48]. Similarly, PF-573228 and PF-431396, dual inhibitors of FAK and PYK2, significantly inhibit cell growth, motility, and invasion while inducing apoptosis in PDAC cell lines [49]. VS-4718 has demonstrated radio-sensitization effects by reducing ECM production and enhancing the efficacy of radiotherapy [50]. Furthermore, VS-4718 has shown significant effects in 3D multicellular models, supporting its potential for clinical use. By reducing ECM production from pancreatic stellate cells, these inhibitors decrease the growth of tumor aggregates in 3D multicellular tumor models [50].

Mechanistically, FAK inhibition has been shown to reprogram cancer-associated fibroblasts (CAFs), suppressing the production of fibroblast growth factor-1 (FGF1), a driver of resistance to RAF-MEK inhibition [51]. Combining FAK inhibitors with Janus kinase/STAT3 inhibition has yielded significant tumor growth inhibition and increased survival across multiple PDAC mouse models, highlighting the potential of dual stromal and tumor-targeting therapies [51]. FAK/AKT/MMP activation causes stress-induced phosphoprotein-1 and contributes to pancreatic cancer progression [52]. Recent studies have also shown that FAK promotes cell proliferation and survival in pancreatic cancer through its interaction with IGF-IR. Thus, downregulating FAK using anti-FAK siRNA increases apoptosis in pancreatic cancer. In addition, cell migration and invasion are reduced by inhibiting FAK with siRNA [17].

## Molecular structure of FAK

**Fig. 1:**
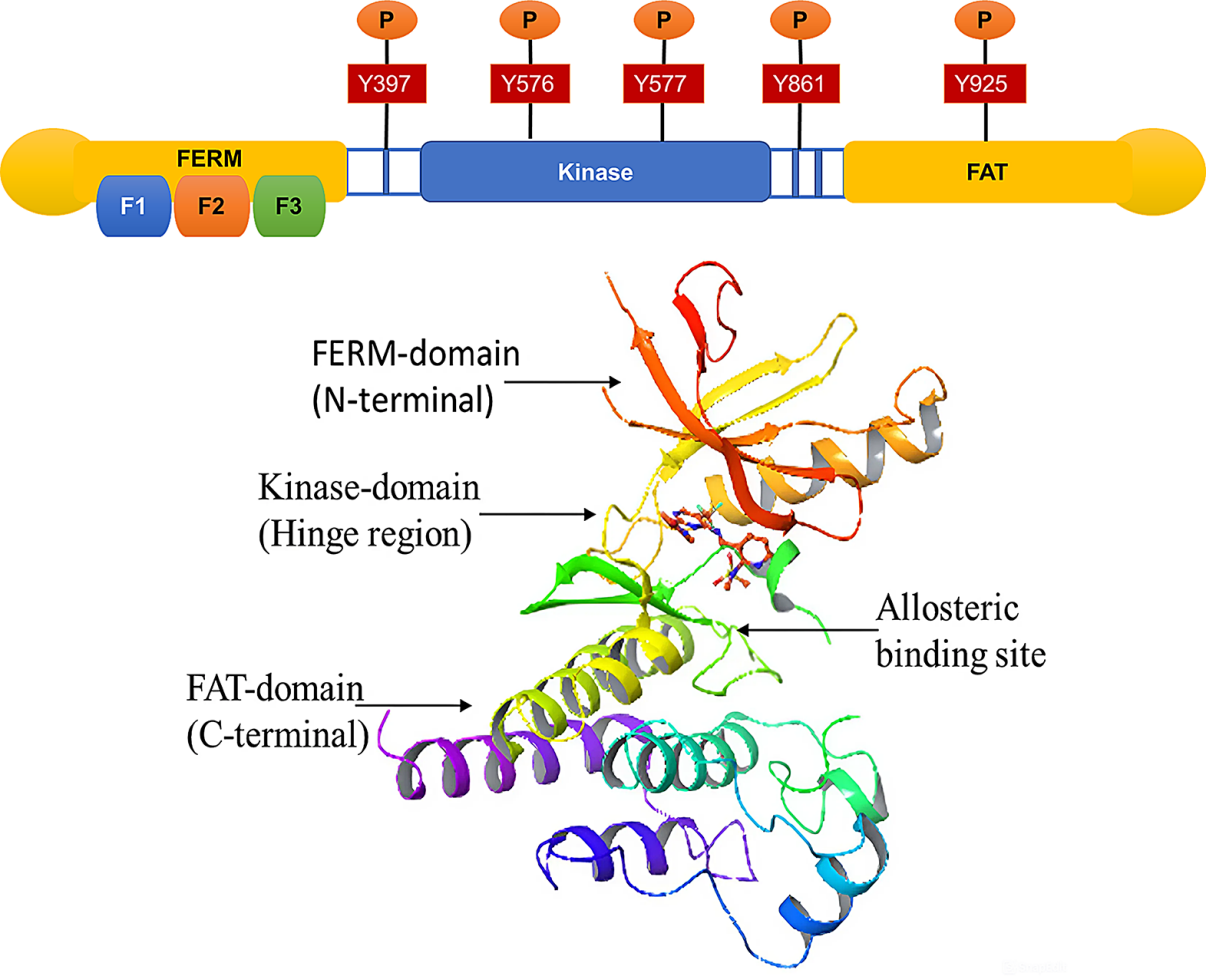
Schematic Representation of FAK: It has three domains; the first is the FERM domain on the N-terminal and consists of autophosphorylation site Y397. The central domain, the kinase domain, and two crucial phosphorylation sites, Y576 and Y577, fully activate FAK and play an essential role in catalytic activity. The FAT domain consists of Y861/Y925, providing a binding site for the SRC complex, which leads to cell survival and translocation signaling. Protein is taken from PDB (3BZ3) and modified in the Schrodinger tool

The FAK is structured into three main parts: the N-terminal, the kinase domain, and the C-terminal region (Fig. 1).

### N-terminal

The FERM domain in the N-terminal is central to FAK’s function, consisting of three lobes, F1, F2, and F3, organized in a clove-leaf-like design. The F1 lobe follows a structure similar to ubiquitin-like folds. It contains five strands of beta-pleated sheets, covered with alpha-helices, which are essential for maintaining the structure and function of FAK. The F2 Lobe subdomain shares similarities with acyl-CoA binding proteins and plays a vital role in regulating FAK. The KAKTLRK patch sequence within F2 is essential for FAK activation through binding with growth factors. This interaction triggers cell stimulation and adhesion. The F3 lobe resembles Pleckstrin Homology (PH) and phosphotyrosine-binding domains. However, it lacks the structural features required to bind acidic phospholipids or phosphorylated tyrosine peptides. Its role is more involved in protein interactions within the FERM domain than lipid binding [53]. The FERM domain is also responsible for FAK’s autoinhibition. It binds to the C-lobe of the kinase domain, preventing FAK activation. This binding inhibits autophosphorylation at Y397 and prevents the recruitment of Src and other SH2 domain proteins, such as paxillin and p130Cas, crucial for FAK’s downstream signaling. This inhibition ensures that FAK is inactive until the proper signals trigger activation. Additionally, the F1 subdomain contains a nuclear localization sequence (NLS), which facilitates the communication of FAK with other proteins, aiding in cellular functions like cell survival by interacting with p53, a key regulator of apoptosis [53].

### Kinase domain

The catalytic kinase domain of FAK is crucial for its enzymatic activity, and its overexpression is often observed in cancer, contributing to tumor progression. This domain contains two key tyrosine residues, Y576 and Y577, whose phosphorylation is essential for fully activating FAK. When these tyrosines are phosphorylated, the kinase domain undergoes a conformational change that enables its catalytic activity. In the inactive form, the activation loop of the kinase domain is disordered, preventing phosphorylation. However, upon phosphorylation of the tyrosine residues, the activation loop forms a beta-hairpin-like structure, essential for enabling protein interactions and the kinase’s function. This structural change is crucial for properly functioning the kinase domain and the downstream signaling pathways that FAK regulates. The C456 and C459 amino acids, which are involved in a bisulfite interaction, likely contribute to regulating the kinase domain. These interactions help stabilize the structure of the kinase domain, playing a key role in its activation. FAK’s kinase domain is inhibited through intramolecular interactions with the FERM domain in its inactive form. The FERM domain physically blocks the activation loop and prevents phosphorylation at Y397, a key regulatory site, thus ensuring that FAK remains inactive until it is triggered by external signals [54]. As a therapeutic strategy, many small molecule inhibitors have been developed to target the catalytic kinase domain of FAK. These inhibitors bind to the kinase domain and block its activity, thereby preventing the downstream signaling that contributes to processes like cell migration, invasion, and survival. One such example is focal adhesion interaction proteins, which can inhibit kinase activity by binding directly to the catalytic domain, rendering it inactive. The targeting of the kinase domain has been identified as a promising approach for cancer therapy, especially in cancers where FAK is overexpressed and contributes to disease progression [53, 54].

### C-terminal

The C-terminal domain of FAK plays a crucial role in regulating its interactions with various binding partners and is key to its function in focal adhesion (FA) signaling. This domain comprises the Focal Adhesion Targeting (FAT) domain and two proline-rich regions. The FAT domain is fundamental as it is a docking site for critical proteins involved in FA formation and signaling, including talin, p130Cas, and paxillin. These interactions are essential for the proper assembly and function of focal adhesions, which are structures that mediate cell adhesion to the extracellular matrix (ECM) and link it to the actin cytoskeleton [53, 54]. Research has aimed to validate the crystal structure of the FAT domain, as understanding its precise structure could enhance our knowledge of how FAK contributes to cellular processes like migration, proliferation, and survival. The relationship between FAT and FERM domains is critical in FAK activation and cellular localization. The FERM domain (located at the N-terminal) is involved in autoinhibition and regulates FAK’s activity, while the FAT domain can modulate the interaction of FAK with other proteins, thus activating or inhibiting various signaling pathways. Additionally, the C-terminal domain contains critical tyrosine residues (Y861 and Y925) involved in trans-phosphorylation, which is essential for FAK’s activation and function. These residues are phosphorylated in response to signaling events, enabling the recruitment of Src family kinases and other downstream signaling molecules that drive cellular processes related to adhesion, migration, and invasion. By interacting with paxillin, the FAT domain also localizes FAK to specific cellular compartments, such as focal adhesions and the nucleus. This localization is critical for coordinating cellular responses to mechanical and biochemical cues from the ECM [53, 54].

In summary, the C-terminal domain of FAK, particularly the FAT domain and the proline-rich regions, regulates the interactions that drive focal adhesion dynamics and signal transduction, with tyrosine phosphorylation (such as Y861 and Y925) further modulating FAK’s activation and interaction with key signaling molecules like Src. These features make the C-terminal domain a critical target for therapeutic strategies to disrupt FAK signaling in diseases like cancer [53, 54].

## Mechanism of FAK activation and inhibition

**Fig. 2:**
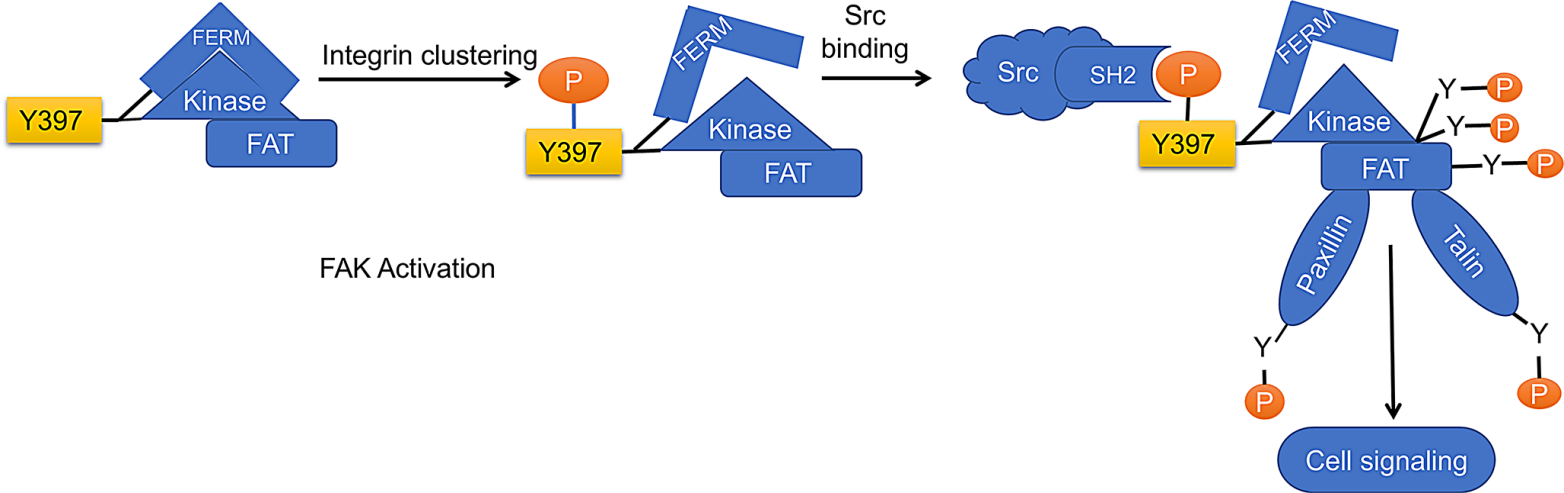
Mechanism of FAK Activation and cell signaling: FAK remains inhibited when FERM is intercommunicated with the kinase domain. Integrin clustering leads to the activation of FAK. Phosphorylation at Y397 leads to the binding of Src-SH2. This leads to the activation of other tyrosine for fully activating FAK and catalytic activity, along with the phosphorylation of other FAK proteins like paxillin and talin. In the case of disease conditions like cancer progression, there will be an alteration in the expression of FAK and signaling in cancer cells

The FAK is a multi-domain protein with three main functional regions: the FERM, kinase domain, and FAT. As shown in Fig. 2, FAK is typically maintained in an inactive, autoinhibited state when the FERM and kinase domains interact intramolecularly, preventing phosphorylation and signaling. This inhibition halts cellular processes like migration, proliferation, and survival [11].

### Mechanism of FAK activation

The activation of FAK occurs when integrin, G protein-coupled receptors (GPCRs), or growth factors stimulate clustering events in the extracellular matrix (ECM). This results in the relief of autoinhibition, allowing FAK to undergo autophosphorylation at Tyr397 in the linker region between the FERM and kinase domains. Phosphorylation at Tyr397 enables the recruitment of the Src-SH2 domain, creating a double-edge complex between FAK and Src kinase. This interaction further promotes phosphorylation of other critical tyrosine residues, such as Y576, Y577, Y861, and Y925, within the activation loop of the kinase domain and between the F1 and F2 subdomains of the FERM domain. The formation of this FAK-Src complex activates a cascade of cell migration, proliferation, and survival, crucial processes for tissue repair, immune response, and cancer metastasis [16].

### Auto-inhibition of FAK

In its inactive state, FAK adopts a closed-conformation, where intramolecular interactions between the FERM domain and kinase domain at Phenylalanine-596 prevent the kinase from being activated. This auto-inhibition ensures that FAK remains dormant until external signals initiate its activation. Additionally, several proteins, such as FAK-interacting protein 200 (FIP200) and Ephrin type-A (EPHA), play a role in maintaining FAK’s inactive state. FIP200 is a large 200 kDa protein that interacts with the FAK catalytic kinase domain, suppressing its activity. FIP200 plays a significant role in cell survival, motility, and adhesion. Studies have shown that the loss of FIP200 can lead to embryonic mortality, as well as severe degeneration and failure in organs like the liver and heart [55].

### Role of FAK-Related non-kinase (FRNK)

FRNK is a 43 kDa protein that is produced as a natural, endogenous inhibitor of FAK. FRNK contains only the C-terminal FAT domain and competes with full-length FAK for binding to the C-domain. By disrupting FAK’s ability to form complexes and engage in downstream signaling, FRNK serves as a competitive inhibitor of FAK activity. Studies have demonstrated that the expression of FRNK inhibits FAK-mediated signaling, leading to reduced metastasis. This makes FRNK a potential therapeutic target in cancer, where FAK is often overexpressed and plays a key role in tumor progression and metastasis [55].

In summary, FAK’s regulation involves complex mechanisms of autoinhibition and activation, with key interactions between the FERM, kinase, and FAT domains. The activity of FAK is crucial for cellular processes like migration and survival, and its dysregulation is linked to various cancers. Inhibitors like FRNK and FIP200 play an important role in controlling FAK’s activity and may be harnessed therapeutically to limit cancer metastasis [16, 55].

## Development of FAK ATP-Competitive kinase inhibitors

Inhibition of FAK can be achieved by targeting different sites, such as the central kinase domain, inhibiting autophosphorylation at a distinct site, or targeting protein scaffolding. Several small molecules bind to the active kinase domain, obstructing FAK’s function and blocking its activity. These ATP-competitive kinase inhibitors are listed in Table 1.

**Table 1:** Kinase inhibitors or ATP-Competitive kinase inhibitors

Compound	Structure*	IC_50_	Reference
PF-573228		5 µM	[56]
PF-562271		1.5 nmol/l	[57]
PND-1186		~ 100 nM	[58]
NVP-TAE 226		6.79 nM	[59]
GSK2256098		1.5 nmol/l	[60]
Defactinib( VS-6063)		0.6 nM	[61]
Ifebemtinib (BI-853520)		1 nM	[62, 63]

*Structures downloaded from ChemDB Chemoinformatics Portal Smi2Depict (https://cdb.ics.uci.edu/cgibin/Smi2DepictWeb.py)

Defactinib (VS-6063) is a second-generation FAK and proline-rich tyrosine kinase-2 (Pyk2) inhibitor that attenuates cell motility, survival, and apoptosis processes Preclinical studies show an IC_50_ value of 0.6 nM in vitro, inhibiting phosphorylation at the Y397 site. This drug is a more specific inhibitor of FAK than Pyk2 [61]. Clinical trials have reported Grade 1 or Grade 2 unconjugated hyperbilirubinemia in patients. A study on pancreatic neuroendocrine tumors (PanNETs) found that overexpression of FAK leads to the cancerous conversion of islet cells and metastasis. Compared to monotherapy, dual treatment with docetaxel and VS-6063 showed improved effects in docetaxel-resistant CRPC cells, increasing apoptosis [64].

Extensive clinical trials have further highlighted defactinib’s potential. Phase I/Ib trials demonstrated that the drug exhibits favorable pharmacokinetics and tolerability, with some patients experiencing stable disease in ovarian, colorectal, and biliary cancers when treated with paclitaxel [65]. Defactinib’s role in cancer therapy has been further reinforced by its FDA Breakthrough Therapy Designation for use in combination with avutometinib for recurrent low-grade serous ovarian cancer (LGSOC) [66]. This designation underscores its potential to significantly improve progression-free survival for patients with this challenging cancer subtype. A pivotal Phase 3 trial (GOG-3097/ENGOT-ov81/GTG-UK/RAMP 301) is currently underway, comparing this combination therapy with investigator-chosen treatments, with results anticipated to provide further insights into its efficacy [66]. Additionally, defactinib has shown promise in other cancers, such as pancreatic ductal adenocarcinoma (PDAC), where it is being investigated in combination with pembrolizumab and gemcitabine [67]. In oesophageal squamous cell carcinoma, defactinib targets the PI3K/AKT pathway to inhibit malignancy [68], and in non-small cell lung cancer (NSCLC), it has shown potential in treating KRAS-mutated tumors when combined with other agents like D-PROTACs [69].

Inhibitor drugs like PF-562271 are methane sulfonamide diaminopyrimidines. X-ray crystallography has shown that PF-562271 binds to the ATP site of FAK, forming an H-bond with the hinge region of the kinase domain. The interaction involves CYS-502 and ARG-426. PF-562271 also interacts with the activation loop region of FAK, establishing connections with residues in the DFG motifs. The antitumor activity of PF-562271 has been tested in breast, pancreatic, lung, and colon xenograft models. The IC_50_ value of this compound is 1.5 nmol/L [70].

Compound PF-573228 is a pyrimidine analog with an IC_50_ ranging from 0.1 to 5 µM and binds to the ATP-hinge region of FAK. Cell culture studies showed that inhibiting catalytic activity and phosphorylation at the Y397 region leads to a lack of inhibition. However, the drug reduces focal adhesion expression. These studies confirmed that PF-573228 inhibits cell growth at high concentrations and may also target kinases other than FAK [71].

VS-4718 (PND-1186) is a pyridine analog and an orally tolerated compound, highly selective for the ATP kinase domain. It has been screened in patients with solid tumors and shows an IC_50_ of approximately 100 nM and a value of 1.5 nM in vitro. PND-1186 has been reported to induce apoptosis in cancer cells. Additionally, inhibition of FAK by PND-1186 leads to anti-inflammatory action by activating caspase-3, reducing tumor growth and metastasis in an ovarian cancer model [72].

TAE226, a pyrimidine analog from Novartis, is a potent ATP-kinase inhibitor that hinders autophosphorylation at the Y397 and Y861 sites, with an IC_50_ of 6.79 nM. In experiments on ovarian cancer cells, TAE226 decreased tumor weight by 54–79% on its own. In a resistant ovarian cancer cell line (Hey-MDR), the compound reduced tumors by 46%. Studies on 37 cancer cell lines showed that TAE226 exhibits a strong anti-proliferative effect, including in multi-drug resistant cells. The investigational compound inhibits cancer cell proliferation and FAK, inhibiting AKT at site S473 through IGF-1R signaling [73].

GSK2256098, a small molecule from GlaxoSmithKline, is a competitive, reversible inhibitor of FAK, targeting ATP kinase activity and inhibiting autophosphorylation at the Y397 site. Its inhibitory concentration is 1.5 nmol/L. This monotherapy is used as an anti-angiogenic agent for advanced solid tumors. A study on 96 cancer cell lines showed sensitivity in glioblastoma cells. Tumor penetration of the drug was notably high, though minimal penetration across the blood-brain barrier (BBB) was observed. The drug blocked tumor infiltration, and combination therapy with cytotoxic drugs reduced tumor weight by 99% in certain models [60].

BI-853520 (ifebemtinib) targets the ATP active site of FAK, with an inhibitory concentration of 1.0 nM in vitro. A study showed that most E-cadherin-deficient cells and lung, ovary, kidney, pancreas, and prostate adenocarcinomas were notably responsive to this drug. While no Grade 4 or 5 adverse events were observed, Grade 3 proteinuria occurred in some cases, and its relationship with the drug remains uncertain, warranting further investigation. In vitro and in vivo studies on breast cancer showed that BI-853520 downregulates FAK expression and decreases cell proliferation. At a concentration of 0.1 µM, it reduces autophosphorylation at Y397 [74].

### Development of FAK allosteric inhibitors

Recent small molecule FAK inhibitors are allosteric site binders that disrupt the communication between FAK proteins without targeting the ATP site, as listed in Table 2. These compounds bind to distinct sites within the kinase domain and are considered inhibitors of FAK. For example, Y11, a novel small molecule (1-(2-hydroxyethyl)-3,5,7-triaza-1-azoniatricyclo-[3.3.1.13,7]-decane bromide), inhibits FAK activity with an IC_50_ value of approximately 50 nM [62]. Targeting the Y397 site on FAK inhibits intracellular signaling, cell proliferation, and survival. Y11 was designed using computer-aided drug design to target the Y397 autophosphorylation site by binding to the N-terminal domain of FAK without altering kinase function. The compound shows a good docking score that satisfies Lipinski’s rules, and the octet binding assay confirms its binding to the N-terminal of FAK [75]. In colon carcinoma models, Y11 reduces tumor growth, induces apoptosis, and activates caspase-3 [76].

**Table 2:** FAK allosteric inhibitors

Compound	CAS Registry Number	Structure*	Reference
Y11	1086639-59-9		[62]
Y15	4506-66-5		[77]
Roslin 2 (R2)	29574-21-8		[78]

*Structures downloaded from ChemDB Chemoinformatics Portal Smi2Depict (https://cdb.ics.uci.edu/cgibin/Smi2DepictWeb.py)

Y15, also known as 1,2,4,5-benzene tetramine tetrahydrochloride, is a novel molecule designed using computer-aided tools and screening small molecules from the National Cancer Institute database. Over 14,000 small molecules were docked with the N-terminal domain of FAK, identifying about 35 compounds that bind to and show strong interactions with the Y397 structural pocket of FAK [77]. Y15 reduces cell viability in all tested cancer cell lines. Western blotting confirmed that it inhibits phosphorylation at the Y397 site and affects downstream factors such as paxillin and Y118-paxillin. An in vitro kinase assay showed that Y15 directly inhibits autophosphorylation without altering overall kinase activity. Compared with TAE226, Y15 caused less apoptosis but significantly inhibited tumor growth in animal models. The in vitro IC_50_ value for inhibiting FAK phosphorylation is 1 µM [77, 79].

Roslin 2 (R2) is a new small molecule that binds to FAK and inhibits its interaction with p53. This interaction was confirmed using in silico techniques. The binding of the p53 N-terminal domain with the FAK N-terminal domain leads to the differentiation of cells. A study on 600 breast cancer tumors revealed a clear relationship between p53 anomalies and the overexpression of FAK, which inhibits p53-induced apoptosis. In cancer cells, p53 is often inhibited, and reactivating p53 could serve as a potential therapeutic strategy. The R2 series of compounds were docked to the FAK and proline-rich regions of p53, as confirmed by computer modeling, and showed a cytotoxic effect in HCT116 cells. R2 was more effective in reducing colon cancer growth when combined with 5-fluorouracil or doxorubicin. It also decreased tumor growth, volume, and the expression of p21 and caspase-3 in vivo. R2 blocks tumor growth by reactivating the transcriptional activity of p53 [78].

## Development of novel PROTACs as FAK inhibitors

Proteolysis-targeting chimeras (PROTACs) are a revolutionary class of therapeutic molecules that utilize the ubiquitin-proteasome system to selectively degrade disease-related proteins. This approach is particularly impactful for targeting proteins previously considered “undruggable,” thereby expanding the range of therapeutic interventions for complex diseases such as cancer [80].

As mentioned earlier, traditional small-molecule inhibitors of FAK primarily target its kinase or allosteric domains, leaving its scaffolding functions intact. However, PROTACs targeting FAK overcome this limitation by degrading the protein entirely, disrupting both its kinase-dependent and kinase-independent roles in cancer progression [81]. Recent advancements in the development of FAK-targeting PROTACs have demonstrated their ability to significantly inhibit tumor growth and metastasis in preclinical models [81, 82]. These molecules have shown superior efficacy compared to traditional FAK inhibitors by reducing cancer cell migration, invasion, and survival. For instance, compound 16b, developed using a defactinib derivative and a lenalidomide analog as its E3 ligase ligand, has demonstrated potent FAK degradation in A549 cells with a DC₅₀ of 6.16 ± 1.13 nM. Further, compound 16b, significantly inhibits cell proliferation, migration, and invasion through a CRBN-dependent proteasome mechanism [83]. Similarly, F2, derived from the FAK inhibitor IN10018, has shown inhibitory activity against several cancer cell lines, reversing multidrug resistance (MDR) by targeting AKT and ERK signaling pathways while reducing P-glycoprotein (P-gp) levels, with IC₅₀ values ranging from 0.73 to 5.84 µM [84].

D-PROTAC and BSJ-04-146 exemplify the enhanced efficacy of FAK PROTACs in targeting cancer cells. D-PROTAC achieved over 90% degradation of FAK in KRAS mutant NSCLC A427 cells at 800 nM, surpassing defactinib in reducing cell viability, migration, and invasion, while achieving an 85% reduction in tumor growth in vivo [69]. BSJ-04-146, with its proteome-wide specificity, enabled rapid and durable FAK degradation, outperforming traditional kinase inhibitors by improving downstream signaling, cell viability, and migration control [85]. Similarly, PROTAC-3 demonstrated superior performance over defactinib in regulating FAK activation, migration, and invasion, showcasing the broader potential of PROTACs to expand the druggable scope beyond traditional small-molecule therapeutics [86]. Among the most advanced FAK PROTACs, A13, built using a PF-562271 derivative as its FAK ligand and pomalidomide as the E3 ligase ligand, exhibited significant FAK kinase inhibitory activity with an IC₅₀ of 26.4 nM. It achieved optimal degradation of 85% at just 10 nM, demonstrating better antiproliferative and anti-invasion effects compared to its parent compound PF-562271, along with excellent plasma stability [87].

However, despite their promise, the clinical translation of PROTACs faces significant challenges. Poor solubility, low cell permeability, and suboptimal pharmacokinetics must be addressed to optimize their therapeutic potential. Hence, designing efficient linkers and delivery systems is crucial for improving the bioavailability and stability of these molecules [88]. As research progresses, strategies like combination therapies may enhance the efficacy of PROTACs, enabling them to work synergistically with existing treatments to target complex oncogenic pathways [88]. Thus, future research and innovation in PROTAC technology hold the potential to unlock new possibilities for combating cancer and improving patient outcomes.

## Development of peptides as FAK inhibitors

Peptides have emerged as promising inhibitors of FAK due to their ability to disrupt protein-protein interactions and modulate cell signaling pathways. These molecules mimic or compete with natural binding partners, providing a precise mechanism to target specific protein interactions. Advances in peptide design have improved their therapeutic potential by enhancing target affinity, selectivity, cell permeability, and resistance to degradation. Overall, peptides have high biological activity, low toxicity, and exceptional specificity and significant chemical diversity, allowing for tailored designs and minimal off-target effects [89]. Several peptides highlight the versatility as FAK inhibitors. The LD2-LD3-LD4 polypeptide competes with endogenous paxillin for binding to the FAK FAT domain. Preventing FAK localization at focal adhesionsisrupts FAK-dependent integrin signaling and scaffolding functions, reducing focal adhesion turnover, tumor cell migration, and invasion [90]. Another promising molecule is the VEGFR-3-derived peptide AV3, a 12-amino-acid sequence paired with a TAT cell-penetrating motif that binds to the FAK carboxylic acid terminus, displacing it from focal adhesions. Its mechanism effectively decreases cell proliferation, induces detachment, and triggers apoptosis in breast cancer cell lines, showcasing its therapeutic potential [91]. Additionally, a 33-residue peptide derived from the F1 lobe of FAK interrupts the FAK-Akt1 interaction, a critical component of cancer cell survival. This disrupted interaction, particularly under increased extracellular pressure, prevents metastasis in murine CT-26 and human Caco-2 cell lines [92]. The disintegrin peptide molecule activates FAK via the integrin signaling pathway. When tested in immunodeficient mice, this peptide delayed the engraftment of Ph + leukemia cells, especially when combined with other treatments, highlighting its potential in combination therapies [93].

Despite their promise, peptides face limited stability, proteolytic sensitivity, rapid clearance, and poor bioavailability. Their gastrointestinal tract instability requires injection administration, and synthesis costs remain high [94]. Thus, addressing these limitations through improved stability, delivery mechanisms, and cost-effective production is essential to utilize their full therapeutic potential.

## Combinational approach to target FAK

Cancer generally arises due to the overexpression of one or more intracellular signaling pathways, and targeting a single pathway may not always be effective [16]. Therefore, combinational therapy, which targets two or more pathological mechanisms, is often used to avoid resistance (Table 3). FAK inhibitors are frequently combined with other agents to enhance therapeutic outcomes.

**Table 3:** Combinational approach to target various types of cancers

Combinational Inhibitors	Target	Disease	Activity	Reference
TAE-226 + docetaxel	FAK	Various types of cancer	↑apoptosis ↓decrease micro-vessel density	[73]
PF-228/PF-271 /defacitinib + docetaxel	FAK	Docetaxel resistant patients, NSCLC, breast cancer, ovarian cancer, Colorectal Cancer, Head and Neck Squamous Cell Carcinoma (HNSCC)	↓phosphorylation of FAK and AKT	[64]
Erlotinib/gefitinib + PF-228/PF-271	FAK and EGFR	NSCLC Ovarian Cancer	↑ apoptosis (-) phosphorylation	[95]
GSK2256098 + trametinib	FAK and MEK1/2	Solid tumors like melanoma, NSCLC, ovarian cancer, HNSCC	Synergistic effects	[96]
CT-707 + cabozantinib	FAK, MET multi tyrosine kinase	Hepatocellular Cancer	↑apoptosis ↑caspase activity	[97]
PF-4554878 + Trastuzumab	FAK and mAb	ER+/HER2 in breast cancer, Esophageal Cancer and Gastric Cancer,	↓phosphorylation of FAK, MAPK, and AKT	[98]
PF-573228 + lexatumumab	FAK and mAb	Pancreatic resistance, Ovarian Cancer, gastric cancer, breast cancer, HNSCC	↑apoptosis, caspase-3, and caspase-8 ↓Bcl-xL ↓mass of tumor	[99]
OXA-11 + sunitinib	FAK and VEGFR-2	Pancreatic neuroendocrine cancer	↓mass of tumor Anti-metastasis`	[100]

In NSCLC, EGFR is overexpressed in 80% of cases, and inhibiting EGFR function with drugs like erlotinib and gefitinib has proven beneficial. Several studies have shown increased FAK expression in NSCLC, which is crucial in cancer progression. To evaluate the efficacy of dual therapy, a study combined FAK inhibitors (PF-228 and PF-271) with the EGFR inhibitor erlotinib. The results showed significantly reduced tumor growth over time, with dual therapy outperforming monotherapy. Specifically, when the A549 cell line was treated with the combination, apoptosis increased sixfold, and phosphorylation of Akt was blocked [95].

In ovarian cancer, the combination of the FAK inhibitor TAE-226 with docetaxel was tested for its effects on cytotoxicity, apoptosis, angiogenesis, and cell proliferation in taxane-sensitive and taxane-resistant cell lines. Preclinical studies in nude mice with HeyA8 tumors showed a 54-79% reduction in tumor load when either TAE-226 or docetaxel was administered alone. However, when the dual therapy was used, tumor load decreased by 89%, and survival rates significantly improved. The dual therapy also resulted in a notable increase in apoptosis and a reduction in microvessel density, indicating a strong effect on angiogenesis [73].

For castration-resistant prostate cancer (CRPC), docetaxel is a first-line treatment. Trials were conducted with FAK inhibitors (PF-00562271, PF-04554878, and defactinib (VS-6063) co-administered with docetaxel to address resistance to docetaxel in CRPC. First-generation FAK inhibitors showed high toxicity by inhibiting CYP3A, the enzyme that metabolizes docetaxel. However, VS-6063 could reverse docetaxel resistance by altering the IC_50_ value. Dual therapy with VS-6063 and docetaxel significantly reduced cancer cell growth compared to monotherapy. In preclinical studies, tumor volume was reduced, and FAK and AKT phosphorylation were markedly decreased in mice [64].

GSK2256098 is a potent FAK inhibitor that blocks autophosphorylation at the Y397 site, while the MEK1/2 inhibitor trametinib also shows promise. The combination of FAK and MEK inhibitors has demonstrated significant anti-carcinogenic activity, with synergistic effects confirmed in animal studies across various cancer types [96].

Cabozantinib, an FDA-approved MET inhibitor for thyroid cancer, also inhibits multiple tyrosine kinases, including RET and VEGFR-2. It has shown activity against hepatocellular carcinoma, but resistance limits its clinical use. Dual therapy combining MET and FAK inhibitors has been shown to enhance anti-carcinogenic activity. In vitro and in vivo studies with CT-707 and cabozantinib demonstrated increased activity against hepatocellular cancer, reducing tumor size and weight. The combination therapy also increased apoptosis in cancer cells, with elevated caspase cascade activity confirmed by western blotting [97].

In breast cancer, the combination of PF-4554878 and trastuzumab was tested against ER+/HER2 + cancer cells. This dual therapy significantly suppressed cancer cell growth, with marked reductions in phosphorylation of Akt (pS473), MAPK (pS202/Thr204), and FAK at site Y397, compared to monotherapy [98].

For pancreatic Cancer, PF-573228, an FAK inhibitor, was combined with lexatumumab, a monoclonal antibody (mAb), to test its effect on chemo-resistant cancer cells. There were high levels of FAK expression (up to 50%) in pancreatic malignancies. The combination therapy induced potent apoptosis, with a reduction in Bcl-xL and increased caspase-3 and caspase-8 activity, confirmed by western blotting. In vivo studies in rats showed a significant reduction in tumor mass, increased apoptosis, and enhanced caspase-3 activation compared to untreated controls [99].

Finally, the combination of FAK inhibitors OXA-11 and VEGFR-2 inhibitors are effective in treating metastasis in pancreatic neuroendocrine cancer. The combination reduced tumor mass, proliferation, and metastasis in the liver of mice. Additionally, the dual therapy decreased tumor vascularity when combined with VEGFR-2 inhibitors such as DC-101 or sunitinib. OXA-11, in combination with cisplatin, potentiated the antineoplastic effect, resulting in decreased cancer cell growth [100].

## Ongoing clinical trials to study FAK-inhibitors

Current clinical trials on FAK inhibitors primarily focus on combination therapies with cytotoxic drugs to evaluate their safety, efficacy, and pharmacodynamics. These studies aim to determine the maximum tolerated doses (MTD), recommended Phase II doses (RP2D), and progression-free survival (PFS), while also assessing adverse events (AEs) and overall patient outcomes.

### Phase I and II trials evaluating FAK inhibitors

A Phase I trial (NCT03875820) explored the combination of VS-6063 (a FAK inhibitor) and RO5126766 (a RAF/MEK inhibitor) in patients with advanced RAS-mutant solid tumors, including non-small cell lung cancer (NSCLC) and colorectal cancer (CRC). The study completed dose escalation in 24 participants, identifying the maximum tolerated dose (MTD) and advancing to Phase II. The second phase will enroll 56 patients to evaluate pharmacodynamics, tolerability, and toxicity, following the NCI CTCAE guidelines.

Another Phase I study (NCT02758587) investigated the combination of defactinib (a FAK inhibitor) and pembrolizumab (a PD-1 inhibitor) in patients with non-small cell lung cancer (NSCLC), mesothelioma, and pancreatic neoplasms. Preliminary results indicate potential therapeutic activity and a favorable tolerability profile. Key outcomes include the duration of response and prevention of tumor progression.

A Phase II trial (NCT03727880) evaluated pembrolizumab with or without defactinib in resectable pancreatic ductal adenocarcinoma (PDAC). This study focuses on modifying the tumor microenvironment to enhance chemotherapy efficacy. Early data suggest improved pathological complete response (PCR) rates and prolonged survival for patients.

### Combination therapies in specific cancers

In patients with neurofibromatosis type 2 (NF2) mutations and recurrent meningiomas, a Phase II trial (NCT02523014) is investigating the combination of vismodegib and GSK2256098. Preliminary data suggest potential tumor growth inhibition and improved progression-free survival over six months.

Similarly, recurrent low-grade serous ovarian cancer is the focus of two ongoing studies. A Phase II trial (NCT04625270) has evaluated the combination of avutometinib and defactinib, demonstrating promising objective response rates and clinical efficacy. Another recruiting study (NCT06682572) is investigating the same combination in Japanese patients, with an emphasis on confirming safety and response rates.

### Completed trials and key results

Several completed trials underscore the potential of FAK inhibitors. In a Phase I study (NCT01138033), GSK2256098 demonstrated a favorable safety profile and established the maximum tolerated dose (MTD) in patients with advanced solid tumors. Similarly, a Phase I/Ib study (NCT01778803) investigated the combination of defactinib and paclitaxel in ovarian cancer, determining a recommended Phase II dose (RP2D) and confirming the anticancer activity of the combination.

For KRAS-mutant non-small cell lung cancer (NSCLC), a Phase II study (NCT01951690) evaluated defactinib, demonstrating improvements in progression-free survival across four cohorts. Additionally, a study (NCT04620330) investigating avutometinib, either alone or in combination with defactinib, in KRAS-G12V and BRAF-mutant NSCLC showed promising efficacy according to RECIST 1.1 criteria.

### Terminated studies

Despite the promising results, some trials encountered challenges. A study combining VS-4718 with chemotherapy in advanced pancreatic cancer (NCT02651727) was terminated due to insufficient data. Similarly, another trial testing VS-4718 in metastatic cancers (NCT01849744) was prematurely ended for similar reasons. Additionally, a study (NCT02372227) combining GSK2256098 and VS-5584 in mesothelioma was discontinued due to toxicity concerns.

### Emerging trials with novel combinations

Ongoing trials are investigating novel approaches. For example, NCT05512208 evaluates the combination of avutometinib and defactinib in gynecological cancers with RAS/BRAF/NF1 mutations, demonstrating encouraging response rates. Additionally, a Phase I study (NCT06739291) is set to assess SIGX1094R monotherapy in advanced solid tumors, with the trial scheduled to begin in December 2024.

These collective findings highlight the potential of FAK inhibitors in combination regimens to improve therapeutic outcomes across various malignancies. Despite challenges in some studies, key successes emphasize their ability to modulate the tumor microenvironment and enhance the efficacy of chemotherapy or immunotherapy.

## Conclusions and future perspectives

FAK inhibitors are currently being utilized in therapy, but their ability to overcome drug resistance remains limited. To address this, ongoing clinical trials investigate dual therapy combinations of FAK inhibitors with cytotoxic or anti-angiogenic drugs. Most of these trials are in Phase II, focusing on determining the maximum tolerated dose, pharmacodynamic activity, and toxicity profiles. Future research aims to identify novel FAK inhibitors with high efficacy and minimal toxicity. Leveraging computer-aided drug discovery represents a promising approach, as drug development requires a multidisciplinary and multimodal strategy.

## Data Availability

No datasets were generated or analysed during the current study.
